# Detection of Pedestrians in Reverse Camera Using Multimodal Convolutional Neural Networks

**DOI:** 10.3390/s23177559

**Published:** 2023-08-31

**Authors:** Luis C. Reveles-Gómez, Huizilopoztli Luna-García, José M. Celaya-Padilla, Cristian Barría-Huidobro, Hamurabi Gamboa-Rosales, Roberto Solís-Robles, José G. Arceo-Olague, Jorge I. Galván-Tejada, Carlos E. Galván-Tejada, David Rondon, Klinge O. Villalba-Condori

**Affiliations:** 1 Unidad Académica de Ingeniería Eléctrica, Universidad Autónoma de Zacatecas, Jardín Juarez 147, Centro, Zacatecas 98000, Mexico; luiscarlosreveles@uaz.edu.mx (L.C.R.-G.);; 2 Centro de Investigación en Ciberseguridad, Universidad Mayor de Chile, Manuel Montt 367, Providencia 7500628, Chile; 3 Departamento Estudios Generales, Universidad Continental, Arequipa 04001, Peru; 4 Vicerrectorado de Investigación, Universidad Católica de Santa María, Yanahuara 04013, Peru

**Keywords:** backward pedestrian detection, reverse camera, convolutional neural networks (CNN), sensors, distances

## Abstract

In recent years, the application of artificial intelligence (AI) in the automotive industry has led to the development of intelligent systems focused on road safety, aiming to improve protection for drivers and pedestrians worldwide to reduce the number of accidents yearly. One of the most critical functions of these systems is pedestrian detection, as it is crucial for the safety of everyone involved in road traffic. However, pedestrian detection goes beyond the front of the vehicle; it is also essential to consider the vehicle’s rear since pedestrian collisions occur when the car is in reverse drive. To contribute to the solution of this problem, this research proposes a model based on convolutional neural networks (CNN) using a proposed one-dimensional architecture and the Inception V3 architecture to fuse the information from the backup camera and the distance measured by the ultrasonic sensors, to detect pedestrians when the vehicle is reversing. In addition, specific data collection was performed to build a database for the research. The proposed model showed outstanding results with 99.85% accuracy and 99.86% correct classification performance, demonstrating that it is possible to achieve the goal of pedestrian detection using CNN by fusing two types of data.

## 1. Introduction

Currently, road accidents worldwide cause the loss of approximately 1.3 million lives annually, according to World Health Organization (WHO) data [1]. In Mexico, 340,415 accidents were recorded in urban areas in 2021, of which 3849 (1.1%) resulted in the death of at least 1 person. The total number of fatalities and injuries in traffic accidents was 86,867 people, of which 4401 (5.1%) lost their lives at the scene of the accident, and 82,466 (94.9%) suffered some injury, according to data from the Instituto Nacional de Estadística y Geografía (INEGI) [2].

Over the years, vehicles have been equipped with technologies aimed at reducing the number of road accidents. Advanced Driver Assistance Systems (ADAS) [3] are a category of intelligent systems that help to improve road safety and reduce the risk of accidents. Vehicles have many sensors and actuators that allow the development of active and passive safety systems, reducing fatalities in road accidents [4]. Within ADAS, there is a subcategory of intelligent systems intended for preventing collisions, such as Automatic Emergency Braking (AEB), Lane-Keeping Assist (LKA), Adaptive Cruise Control (ACC), Forward Collision Warning (FCW), and Cross-Traffic Assist (CTA) [5].

FCW and AEB are two systems that can work simultaneously and use sensors, LiDAR, and cameras to perform forward detection of vehicles or objects. In various cars, the AEB system has been proven to be effective by using a combination of sensors, LiDAR (Laser Imaging Detection and Ranging), cameras, and software to brake the vehicle when detecting objects, including pedestrians. A subcategory of AEB is Autonomous Emergency Braking Pedestrian (AEB-P), specifically focusing on frontal pedestrian detection [6,7]. This intelligent system avoids collision with pedestrians and uses a combination of parameters, such as collision time, speed, detection time, and AI, to improve road safety.

Pedestrian detection plays a crucial role in reducing traffic accidents due to collisions. Despite this, current approaches to pedestrian detection using AI are based on front-end detection (AEB-P), which involves the usage of cameras and sensors located at the front of the vehicle. The backup camera and sensors at the car’s rear are essential to avoid collisions at the back of the automobile. In 2018, these devices were established as mandatory by law in the United States [8]. In Mexico, since 2020, all new vehicles manufactured domestically or imported must include acoustic and visual warning systems for the driver when the presence of objects or persons behind the vehicle is detected while reversing [9].

The automotive industry is being transformed by AI, which is currently used to create intelligent systems such as autonomous driving [10], AEB-P, FCW, and parking assistants [11,12], among others. AI tools, such as machine learning, computer vision, and deep learning, have provided a set of algorithms that enable the design, development, and improvement of intelligent systems in the automotive industry. In pedestrian detection, AI plays a crucial role. In particular, using CNN [13] has made frontal collision avoidance systems increasingly sophisticated and robust. An example of this is the research conducted by Liu et al. [14], in which CNNs were employed for rain removal, and two modules were proposed: one for rain removal and another for pedestrian detection once the image was regenerated (output of the previous module). In another study [15], the authors used the YOLOv3 CNN architecture to detect pedestrians and vehicles in different scenarios. These scenarios included daytime and nighttime conditions, extreme weather, and even images with a high saturation of people or objects. In addition, several types of research focused on pedestrian detection in low illumination conditions [16,17,18].

Within the literature, numerous studies delve into pedestrian detection in front of vehicles. However, the detection of pedestrians to the rear of cars needs to be further addressed. This lack of attention is due to the limited research conducted in this area. Some notable work in pedestrian forward detection is, for example, the study by Li et al. [19]. Their research presented two innovative methodologies that leveraged the You Only Look Once (YOLO) framework: VggPrioriBoxes-YOLO and MNPrioriBoxes-YOLO. These methodologies were designed to improve peat detection, especially in adverse weather conditions with low visibility. The results obtained demonstrated an average accuracy (AP) of 80.5% for VggPrioriBoxes-YOLO and 80.5% for MNPrioriBoxes-YOLO, which, when compared to other existing methods, showed that the proposed MNPrioriBoxes model performed better in both accuracy and processing speed, allowing efficient pedestrian detection in foggy weather conditions. Also, Tumas et al. [20] presented an infrared imaging forward-pedestrian detection system, which modified the YOLOv3 architecture and used CAN bus data (driving speed, brake pedal status, and temperature) to create an ADAS system to perform advanced predictions in pedestrian time. This approach achieved a mean Average Precision (mAP) of up to 89.1%.

On the other hand, Yi et al. [21] proposed a real-time forward-pedestrian detection algorithm based on Tiny–YOLOv3. They used *K*-means clustering on the training image set to find the best prior features. In addition, three convolutional layers were added to the original network to improve the model’s ability to extract pedestrian features, obtaining an AP of 73.98% with a detection speed of 4.84 ms. Similarly, Zhang et al. [22] proposed an improved LeNet-5 CNN in their research for forward-pedestrian detection. This model met the accuracy and real-time requirements of advanced assisted driving. First, they analyzed the structure of the LeNet-5 network model and improved and optimized the network parameters and design to obtain a new LeNet model used to detect pedestrians. Finally, they obtained a failure rate of 25% in the improved LeNet CNN and a detection speed of 0.204 frames per second. To address these challenges, several methods have been proposed in the literature. Flores Calero et al. [23] presented a Histogram of Oriented Gradients (HOG)-based classifier together with Support Vector Machine (SVM) and Inference Logic (IL) algorithms to discriminate between the person to be detected and the background. They built an alternative dataset to make the experiment more realistic, hiding certain image parts at different percentages of synthetic occlusion (0, 10, 20, 30, and 40 percent). The results showed Area Under the Curve (AUC) values of 99.01%, 98.89%, 98.39%, 97.15%, and 95.41%, respectively, for the different percentages of synthetic occlusion, with a processing speed of three images per second.

In addition, Chi et al. [24] proposed an effective and efficient detection network for detecting pedestrians in crowd scenes. They introduced a mask-guided module to leverage the primary information to improve learning in the ResNet50 CNN architecture, which they used as a backbone. The authors showed the results as the log-average miss rate ($MR^{-2}$), obtaining values of 43.53% and 45.31% in the CityPersons and Caltech-USA dataset, which were lower than other methods used in the literature. Finally, Yang et al. [25] proposed a Part-Aware Multi-Scale Fully Convolutional Network (PAMS-FCN) to address the difficulties of occlusion and small scale when detecting pedestrians. For this, they developed a Region of Interest (ROI) clustering module to extract body parts, which allowed a partially visible pedestrian instance to receive a high detection confidence score, meaning it was less likely to become a false detection. In the Caltech dataset, the proposed network obtained 4.9% in $MR^{-2}$, in addition to presenting competitive detection performance, e.g., 78.43%, 67.20%, and 61.88% concerning AP in easy, moderate, and difficult subsets, respectively, and a detection time of 0.25 s per image.

Furthermore, in [26], Xie et al. proposed a new forward-pedestrian detection model called PSC-Net, which used a CNN to learn the spatial co-occurrence of pedestrian body parts. They achieved absolute gains of 4.0% and 3.4% over Graph Multi-Attention Network (MGAN) on the CityPersons and Caltech test sets, respectively. The research mentioned above focuses on detecting pedestrians under various conditions, such as adverse weather, occlusion, low image quality, small-scale pedestrians, and real-time scenarios. Han et al. [27] proposed a CNN based on Faster R-CNN for small-scale forward-pedestrian detection. The architecture generated more effective ROIs for small-scale pedestrian detection, achieving an AP of 90.51%, slightly outperforming architectures such as YOLOv3 and YOLOv2 with 89.77% and 71.53%, respectively. Meanwhile, Luo et al. [28] proposed RT-YOLOv3, an improved version of YOLOv3, for real-time forward-pedestrian detection at different scales. The proposed method achieved a precision of 93.57% mAP and 46.52 f/s, meeting the requirements for real-time pedestrian detection. Regarding accuracy, RT-YOLOv3 outperformed Fast R-CNN, Faster R-CNN, YOLO, SSD, YOLOv2, and YOLOv3.

Pedestrian detection is a critical issue in road safety, and significant progress has been made in pedestrian detection with cameras and sensors at the front of the vehicle. However, pedestrian detection at the vehicle’s rear remains challenging, especially in urban areas with many pedestrians and parked cars. Backward pedestrian detection is crucial to avoid collisions. In the research by Keall et al. [29], focusing on the effectiveness of rearview cameras and rear parking sensors in preventing pedestrian rearview injuries, the authors provided fundamental insights into the use of these types of tools in crash prevention, as the use of rearview cameras has reduced rear-end collision crashes by 41%. Furthermore, their study showed that the combined use of cameras and sensors alone did not have a statistically significant effect. Within the research oriented toward pedestrian detection in the rear of vehicles, the work by Tadjine et al. [30] focused on the pedestrian identification process. In this context, a fisheye-type camera, placed at the vehicle’s rear, was employed to acquire images of the surrounding scene. The basis of its methodology lay in an algorithm aimed at detecting moving objects, which relied on an adapted variant of the windowing technique used to generate assumptions about the location of the things in question. Subsequently, an analysis of HOG-derived features was implemented in conjunction with an SVM-based classifier.

Another relevant research contribution, documented in the literature, comes from the work of Wang et al. [31]. These authors proposed an approach focused on the safety of heavy-duty trucks during the backing maneuver. Their method used pedestrian detection and obstacle tracking using binocular fisheye cameras. The aforementioned system consisted of four main stages: calibration and image matching of the cameras, distortion correction and pedestrian identification, obstacle tracking through an improvement of the Consensus-based Matching and Tracking (CMT) algorithm, and a procedure for truck speed regulation.

In addition to the research, as mentioned earlier, it has been shown that implementing in-vehicle acoustic and visual alerts has proven effective in avoiding rear-end collisions. Therefore, it is essential to research and develop solutions for in-vehicle pedestrian reversing detection to improve road safety and reduce accidents. Using AI and computer vision techniques, such as CNNs, could be an effective solution to address this problem. To approach this issue, in this research, AI techniques are used, specifically CNNs, which have proven to be very effective in detecting objects in images. By using these techniques, the aim is to achieve a robust and accurate system that can detect pedestrians at the vehicle’s rear and alert the driver of their presence. The results of this research are expected to contribute significantly to road accident prevention and safety.

The paper is structured as follows. In Section 2, the materials and methods employed to carry out backward-pedestrian detection are described, including data acquisition, implementation of CNNs, and validation metrics used. The results obtained using the proposed methodology are presented in Section 3. Afterward, in Section 4, the results obtained in the previous section are looked over to highlight the contribution of this research in comparison with the state of the art. Finally, Section 5 presents conclusions and future work to improve the pedestrian detection system at the vehicle’s rear.

## 2. Materials and Methods

**Figure 1:**
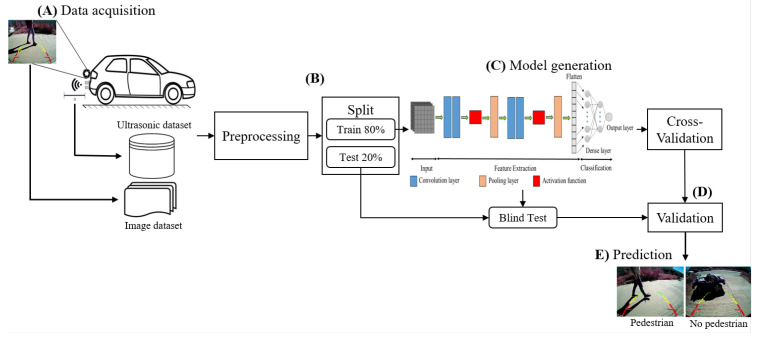
Flowchart of the proposed methodology for pedestrian detection in reversing vehicles. (**A**) Data acquisition system and dataset generation. (**B**) Preprocessing and division of data into two datasets (Train and Test). (**C**) Generation of the proposed model using the datasets. (**D**) Validation of the model to assess its performance. (**E**) Prediction stage.

Figure 1 shows the proposed method for performing backward-pedestrian detection of the vehicle. First, collection and storage needs to be carried out, using the information from the reversing camera and ultrasonic sensors (Figure 1A). In Figure 1B, the data are cleaned and labeled into two sets: Train and Test. Once the corresponding Train and Test images and distances are obtained, in Figure 1C, a model is generated using the Inception V3 architecture to analyze the images. Then, a one-dimensional (1D) CNN architecture examines the distance data from the sensors. In Figure 1D, an evaluation of the performance of the proposed model is implemented by using the following statistical metrics: accuracy, precision, sensitivity, specificity, F1-score, and AUC. Finally, the predictions are carried out, showing the output as pedestrian or non-pedestrian. The steps above are described in detail in the following subsections.

### 2.1. Data Acquisition

Several vehicle models are now equipped with rearview sensors and cameras. Since 2018, a law in the United States has required all vehicles to be fitted with this technology. This technology has proven effective in reducing accidents, although cases of collisions with objects, people, and pets are still reported.

The information provided by reversing cameras and sensors is limited by vehicle manufacturers, making it impossible to access and manipulate this information to develop a system to prevent collisions with pedestrians. Therefore, due to the lack of reverse sensor and camera datasets, a data acquisition system was developed for this research to create an in-house database to detect pedestrians at the rear of the vehicle.

This subsection details the hardware and software used to perform this work and acquire the datasets. The data acquisition was performed by simulating everyday driving actions when reversing the vehicle, having parking lots, garages, and streets as scenarios.

**Figure 2:**
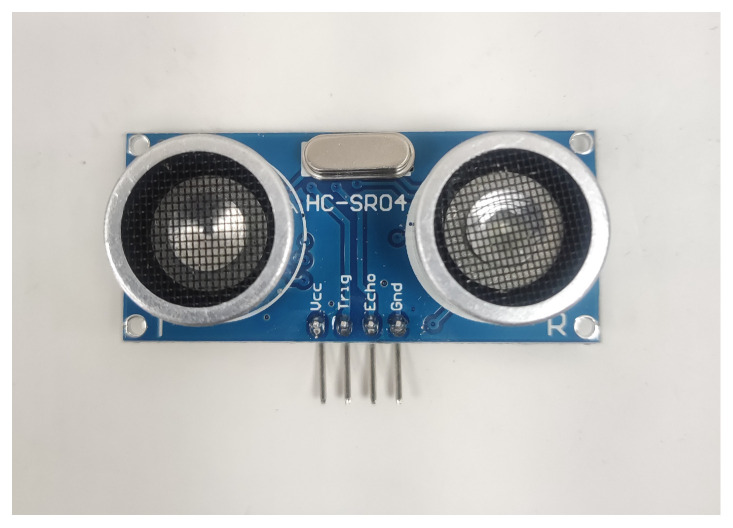
HC-SR04 ultrasonic sensor.

On the hardware side, the processing unit consisted of an Intel i7-8750H at 2.20 GHz (Intel Corporation, Santa Clara, CA, USA), 16 GB of RAM, an NVIDIA GeForce GTX 1050 graphics card (Nvidia Corporation, Santa Clara, CA, USA), as well as input peripherals; four HC-SR04 (see Figure 2) ultrasonic sensors (Cytron Technologies, Simpang Ampat, Malaysia); Arduino 101 (Arduino, Ivrea, Italy); and a commercially available ZHAOCI model 8L backup camera (Paolly, China), which is compatible with any car.

**Figure 3:**
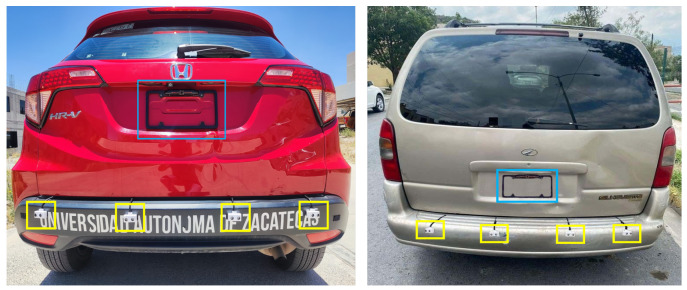
Experimental setup. The blue box = reverse camera installed, yellow boxes = ultrasonic sensors.

For the data acquisition, the camera and the four sensors were installed on the back of two test vehicles, a Honda HR-V (Honda Motor Company, Tokyo, Japan) and a Chevrolet Silhouette (Chevrolet, Detroit, MI, USA), as shown in Figure 3. The camera was calibrated, so that the image view provided was equal to a vehicle with a factory-installed backup camera. The sensors were connected via UTP cable to the GPIO pins on the Arduino 101. The image capture was synchronized to the distance readings of the ultrasonic sensors, obtaining 20 FPS and 20 sensor readings.

**Figure 4:**
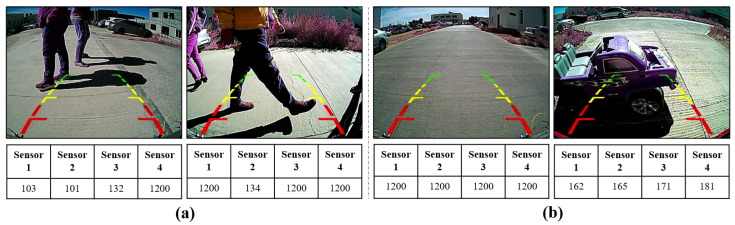
Data types: (**a**) the presence of pedestrians with their respective detection distances, and (**b**) the absence of pedestrians and the presence of objects.

A Python and Arduino program was run simultaneously on a laptop while the vehicle was moving in reverse, collecting data, as shown in Figure 4, obtaining images and distances in **centimeters** from the sensors simultaneously. Figure 4a shows some examples of images containing pedestrians, along with the corresponding distance measurements obtained by the sensors. In contrast, Figure 4b shows images without pedestrians, highlighting the distances when no pedestrians or objects are detected in the presence of items. In particular, it is interesting to note that Figure 4a,b shows values of 1200 in the sensor readings. This observation is because the sensors detect neither objects nor pedestrians.

This research was conducted within the facilities of the Universidad Autónoma de Zacatecas, Campus XXI, utilizing the parking lots, internal roads of the campus, and the exit of the Centro de Investigación Automotriz de México (CIIAM) garage for data collection. The flow of students served as a reference point, acting as a test scenario for the data collection process.

### 2.2. Preprocessing

Currently, pedestrian detection focuses on the front of vehicles using images captured by cameras, and detection is performed using machine learning techniques, specifically, 2D CNNs [32]. However, in this study, the scope is pedestrian detection at the rear of the vehicle by using combined 2D and 1D CNNs [33,34], which allow us to analyze two types of data and increase the accuracy of pedestrian detection when reversing the vehicle. Since there is no suitable public dataset for this purpose, in-house data acquisition was conducted to train and evaluate our proposed model.

During the acquisition, 75,440 images were obtained with their corresponding distance data, resulting in two datasets: the image dataset and the distance dataset of the ultrasonic sensors. However, these data were stored in a random manner, so the data were divided according to the type of image (with a pedestrian, without a pedestrian, or an object); for this, a Python code was coded to facilitate the division of the data into two types: with pedestrians and without pedestrians. Once the dataset of pictures divided into two classes was obtained, the dataset of distances was divided according to the ID of the image, which was assigned at the time of collection; this ensured the distance corresponding to each image. Subsequently, the cleaning of the data was carried out; in other words, an inspection of the pictures with pedestrians was made, ensuring that a distance recorded by any of the sensors was found; otherwise, the image and the corresponding line in the dataset of ultrasonic sensors were eliminated, since these were considered as noise in our data.

Finally, the result of the previous process derived two datasets with 6981 elements in each class, i.e., with pedestrians and without pedestrians, having a total of 13,962 images and 13,962 observations in the dataset of the distances. The datasets were divided into two sets: Train (80%) and Test (20%). The above classes were coded with “1” and “0”, respectively. Since this is a classification problem between two types, the binary cross-entropy loss function was used. This function is appropriate when the objective is to classify between two different classes represented in binary form.

Loss functions play a fundamental role in evaluating the performance and behavior in deep learning models during the training process. In this context, the binary cross-entropy loss function focuses on quantifying the error between the actual class value (1 or 0) and the prediction generated by the model. The result of this function oscillates between a positive value of 0 and 1. When the result is close to 0, it is interpreted that the model presents a low error level in the prediction of the classes, indicating a good learning process. In the other hand, values close to 1 show a significant error, denoting poor learning by the model [35,36].

### 2.3. Model Generation

**Figure 5:**
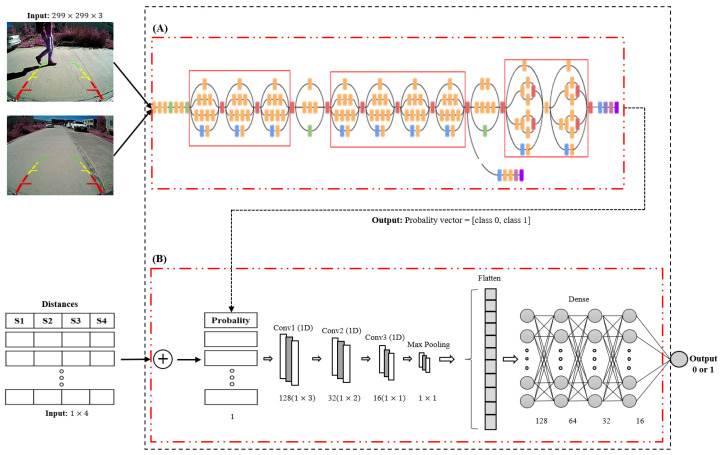
Proposed model for vehicle backward-pedestrian detection, (**A**) Inception V3 and (**B**) 1D model.

For this research, we combined two types of CNNs; a 2D-CNN and 1D-CNN were used to generate the model, as shown in Figure 5. The model consisted of using the Inception V3 architecture [37] and a proposed 1D-CNN architecture, as demonstrated in Figure 5A,B. The architectures of Inception V3 and 1D-CNN were combined into a single block, thus having two inputs to analyze: images and distances, taking as reference two different data types and outputs of our model (“pedestrian” or “non-pedestrian”).

CNNs are designed to process data in three types of dimensions: vector (1D), matrices (2D), and tensor (3D). Popularly, CNNs are used in image and video processing, Natural Language Processing (NLP) [38], and recommendation algorithms. Four critical ideas behind CNNs take advantage of the properties of the inputs: local connections, shared weights, clustering, and the use of multiple layers [39]. CNNs, regardless of input data types, are composed of different filters/cores. These constitute a set of trainable parameters that can spatially convolve the data to detect features [40].

**Figure 6:**
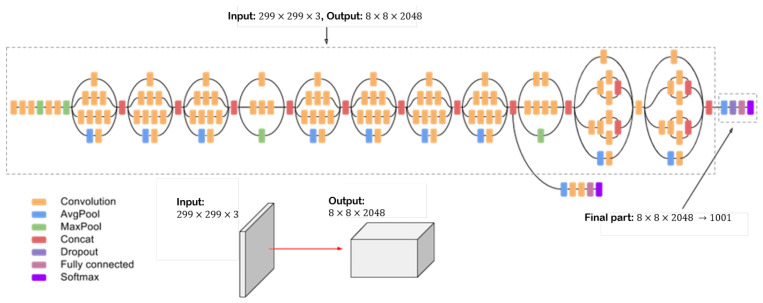
Inception V3 architecture.

Several pre-trained 2D-CNN architectures were established in the literature. Inception V3 is a deep convolution network widely used for classification tasks. It has multiple symmetric and asymmetric building blocks. Each block has several branches of convolutions, average pooling, max pooling, concatenated, dropouts, and fully connected layers [41]. This network has 42 total layers and 29.3 million parameters. Figure 6 shows the architecture of the Inception V3 network [37,42].

The function of Inception V3 in this research is given in the analysis of images with and without pedestrians, providing, as a result, the prediction probability (0.5<P≤1) of finding a pedestrian in the analyzed image. For this, the Train (80%) dataset was used to retrain Inception V3, adding 1000 epochs for training as well as preprocessing the image, then performing a rescaling to 299×299 pixels and a normalization of the image using Equation (1) before training:

(1) $$Img_{norm}=\frac{Img-V_{px_{min}}}{V_{px_{max}}-V_{px_{min}}},$$

where the variables are as follows:

- $Img_{norm}$: corresponds to the normalized image;
- Img: matches the original image;
- $V_{px_{min}}$: the minimum image pixel value; and
- $V_{px_{max}}$: the maximum image pixel value.

Notably, 1D-CNNs are commonly used to analyze one-dimensional signals (vectors, time signals, etc.) and have been implemented in various applications, such as health, brainwave analysis, traffic, marketing, and network analysis. In contrast to 2D-CNN applied mainly to image analysis, one of the distinguishing features of 1D-CNN is the absence of a predetermined kernel size. However, the kernel size is a fundamental parameter that profoundly influences the efficiency of signal analysis. Its determination depends on the dimensions of the input data. Consequently, the absence of fixed kernel size in 1D-CNN generates complexities since optimal kernel specification requires contextual sensitivity, making establishing a universal fixed size impractical [43].

**Figure 7:**
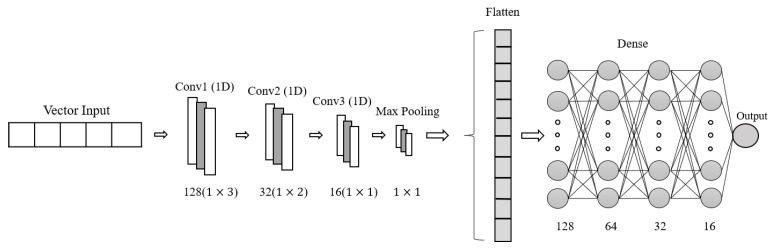
Proposed 1D-CNN architecture.

In this research, a 1D-CNN architecture was built for the analysis of proximity sensor signals, as shown in Figure 7, having, as input, a 1×5 vector, where the first four columns correspond to the distance of the sensors (S1,S2,S3,S4) and the last column corresponds to the probability coming from the analysis of the image. This probability is obtained using Inception V3 for image analysis by calculating the possibility of the presence of pedestrians in the input image. The 1D-CNN architecture also consists of three convolution layers with filters of 128, 64, and 32 and kernels of sizes 3, 2, and 1, which were set according to the shape of the input vector to the model. Four dense layers of sizes 128, 64, 32, and 16, respectively, and an output neuron with the “sigmoid” activation function were added. This is how our 1D-CNN architecture was formed.

Similarly, training was performed with the Train dataset (80%) of the distance dataset, applying 1000 epochs and the 1D-CNN. As mentioned above, we used the binary cross-entropy loss function in this research, as shown in Equation (2) [36,44]:

(2) $$L\left(y,\hat{y}\right)=\left(-\frac{1}{N}\right)\sum_{i=0}^{N}\left(y\times \left(log\left(\hat{y}\right)\right)+\left(1-y\right)\times \left(1-\hat{y}\right)\right).$$

In this equation, the terms plays the following roles:

- *y*: Represents the classes, where, in this context, it takes the value of 1 or 0, according to the label assigned to the image;
- $\hat{y}$: Corresponds to the probability the model has calculated for the class to which the probability prediction is made;
- *N*: Indicates the total number of observations, which, in this specific work, equals 13,962.

This loss function captures the discrepancy between the model predictions and the actual classes on a weighted basis for each epoch in the model training [36]. This approach allows the evaluation of how the model predictions deviate from the actual values as a function of the probability assigned to each class.

### 2.4. Validation

Validation consists of evaluating the model’s ability to predict based on previous training, using different statistical metrics such as the confusion matrix and the Receiver Operating Characteristic (ROC) curves [45,46].

A confusion matrix is a tool that allows the visualization of the performance of an AI algorithm [47,48]. One of the advantages of confusion matrices is that they provide general information about the model’s predictive ability. The information provided shows four widespread terms: True Positive (TP), the favorable cases that the model correctly classifies as positive, that is, the model predicts the class “positive” (label “1”), and the actual title is also “positive” (brand “1”); True Negative (TN) represents the negative cases the model correctly classifies as unfavorable, that is, the model predicts the “negative” class (label “0”); False Positive (FP) corresponds to the negative cases incorrectly classified as positive; and False Negative (FN) refers to the positive cases incorrectly classified as negative. These terms are fundamental for calculating various evaluation metrics and provide information on the model’s ability to discern between the different classes [48]. These terms are essential in the evaluation of classification models. They are used to calculate various metrics, such as accuracy, precision, sensitivity, specificity, and AUC, which provide information on the model’s performance and ability to correctly classify positive and negative instances.

Accuracy corresponds to the proportion of correctly predicted classifications out of the total number of instances [41,49], as shown in Equation (3):

(3) $$\mathrm{Accuracy}=\frac{TP+TN}{TP+TN+FP+FN}.$$

Another evaluation metric is precision; this measure, also known as “predictive value”, evaluates the model’s predictive power. In other words, it is how accurate the model is of those predicted positives and how many are positive, as shown in Equation (4):

(4) $$\mathrm{Precision}=\frac{TP}{TP+FP}.$$

Additionally, it is necessary to know how many positive cases are being correctly classified and how many negative cases are being correctly classified. For this purpose, two metrics are calculated to provide this information. These metrics are called sensitivity (recall) and specificity, and are calculated using Equations (5) and (6), respectively:

(5) $$\mathrm{Recall}=\frac{TP}{TP+FN},$$

(6) $$\mathrm{Specificity}=\frac{TN}{TN+FN}.$$

In addition, for this research, we used the ROC curves, from which we obtained the AUC, which is a metric that indicates high diagnostic accuracy when values are close to 1.0, as calculated by Equation (7). Therefore, using this index is recommended as a general measure of the differences between two classes, achieving accuracy in prediction [50].

(7) $$\mathrm{AUC}=\frac{\mathrm{recall}-(1-\mathrm{specificity})}{2}.$$

### 2.5. Cross-Validation and Blind Test

Based on the above, we perform two validationsed considering two metrics: the *K*-fold Cross-Validation (CV) [51,52,53] and the Blind Test (BT). The *K*-fold CV consists of randomly dividing the Train dataset into *K* samples, where the training and the validation of the generated model are performed for *K* iterations, taking sub-datasets, in this case, K−1, to train the model and K−(K−1) to validate the model. *K*-fold CV is one of the validation methods that helps to verify the model’s learning capacity and to prevent overfitting of the model [51]. Therefore, *K*-fold CV provides a more rigorous evaluation of the model by using all training data samples to train and validate. In contrast to *K*-fold CV, the BT uses the data reserved for testing, which corresponds to 20% of the data set. This test is considered “blind” because the model has had no prior contact with these data. This provides more accurate validation by exposing the model to new data, which is essential for evaluating its performance in real situations.

### 2.6. Prediction

Finally, after training and validating the proposed model, the prediction is performed, testing the model by entering input data (image and distances) and predicting if there is the presence of a pedestrian, resulting in a “1” if so, and a “0” otherwise.

## 3. Results

As mentioned above, a total of 75,440 datapoints were obtained in the data acquisition, from which, later, with the preprocessing, a final sample of 13,962 datapoints was obtained, which then was divided into two different datasets: images and distances. The distances dataset consisted of 13,962 observations and four features, where the corresponding label was added to the data: “1” in the presence of a pedestrian and “0” in the absence of pedestrians. This was the model output, producing a dataset of 13,962 ×5.

The architectures used in this study were trained to consider the training hyperparameters corresponding to each model, as detailed in Table 1. It should be noted that the hyperparameters for the 1D network architecture were selected through an experimental process, and the values presented in Table 1 reflect the most optimal results obtained during this process.

**Figure 8:**
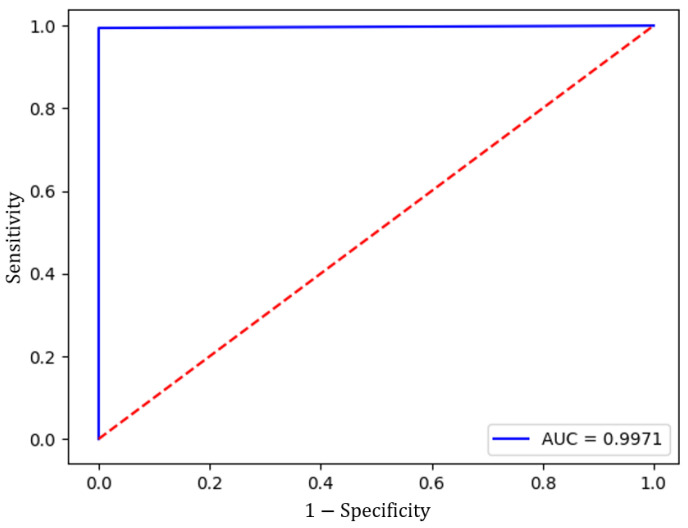
Inception V3 ROC curve. The red line in the figure represents a reference point. The AUC of this linear is 0.5.

The Inception V3 architecture has the function of analyzing the image database, for which the model was trained with 80% of the set of images corresponding to 11,168 prints, 5584 for each class (pedestrian, non-pedestrian), and validated with the remaining 20%, which corresponds to 2794 elements. It is worth mentioning that the result of this analysis has, as output, a probability between 0 and 1, which is concatenated to the distance database to be analyzed with the one-dimensional network architecture. The result is shown in Figure 8, representing the ROC curve of Inception V3. The graph shows that the AUC is close to 1.0, demonstrating CNN’s ability to classify images with and without pedestrians.

The objective of this research is pedestrian detection by merging two data types. This work aims to contribute to a new method to reduce pedestrian collisions when the vehicle reverses. The results of the validations applied in this research are shown as a follow-up.

### 3.1. Result Cross-Validation

Once the model shown in Figure 5 was built, the *K*-fold cross-validation was performed, considering a size of K=5, using 80% of the data, i.e., the portion that made up the training data set, and 1000 epochs to train. The results obtained are shown in Table 2.

**Figure 9:**
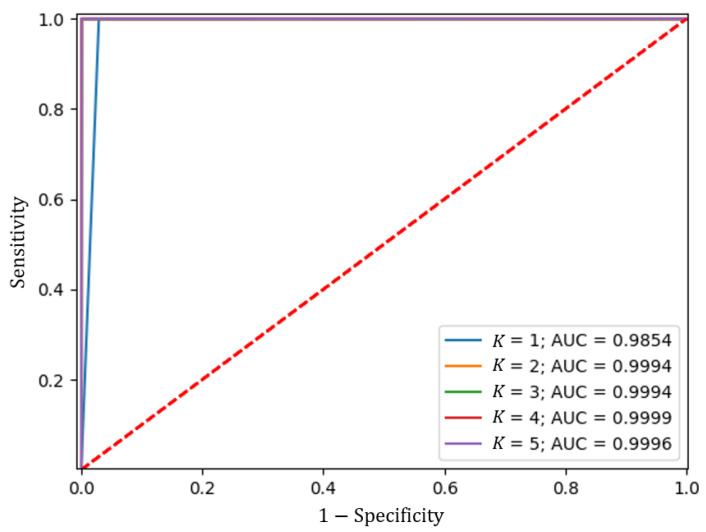
ROC curve of the cross-validation of the proposed model with *K*.

Likewise, the ROC curve was calculated, as shown in Figure 9, from which the AUC shown in Table 2 was calculated.

The above results show robust and consistent performance across all partitions of the Train dataset, having a mean of the metrics close to a unit value. These results indicate that the model can generalize well to unseen data and has a low risk of overfitting.

### 3.2. Blind Test Results

**Figure 10:**
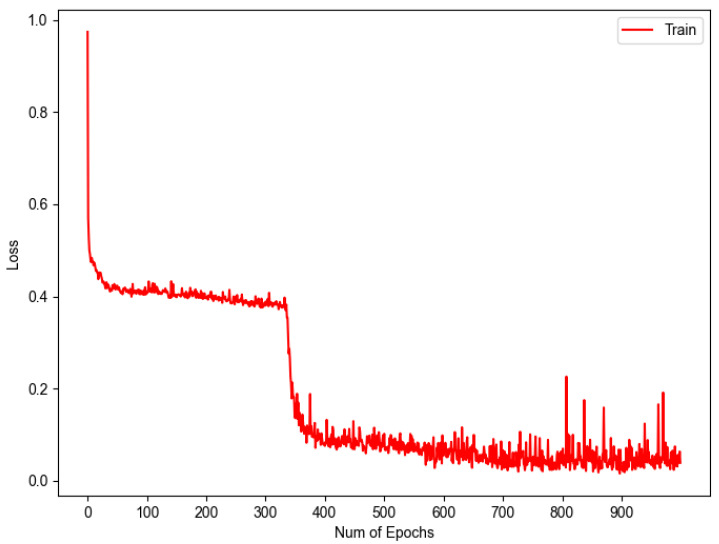
Graph of model training loss.

To evaluate the performance of 1D-CNN against completely unknown data, a blind test was performed using the remaining 20% of the data. The results of the blind test provide a more reliable assessment of the actual model performance, as they are not affected by the model’s fit to the data used during training. For training in this test, 80% of the data reserved for this task were used, generating two plots showing the learning progress over epochs. The initial graph, presented in Figure 10, illustrates the result derived from the application of Equation (2), which leads to the representation of the loss throughout the training process. It is observed that the acquired values are near the threshold of 0, indicating a satisfactory level of learning by our model.

**Figure 11:**
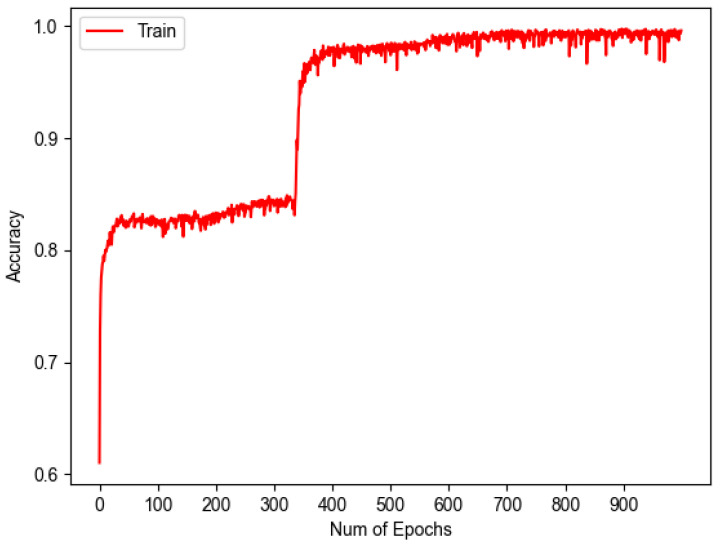
Accuracy graph in model training.

Complementary to Figure 10, the following graph was obtained, which can be seen in Figure 11, representing the accuracy vs. epochs during training.

These two graphs show that as the loss decreases, the accuracy increases, demonstrating that the model is learning properly and proving that these two metrics can be inversely related.

**Figure 12:**
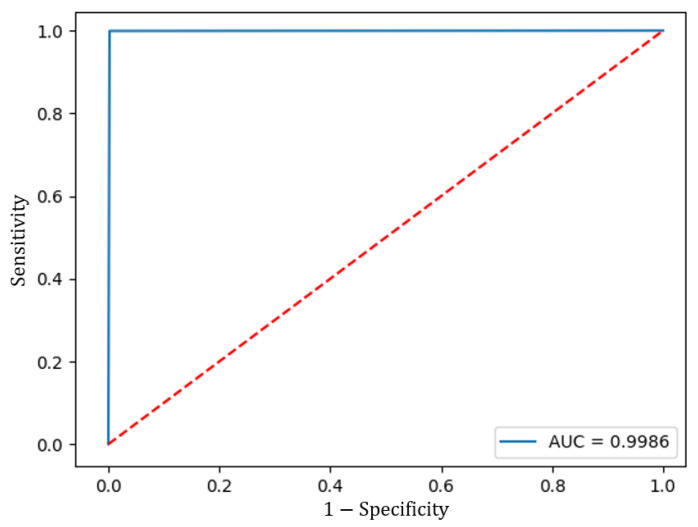
ROC curve of the proposed model.

Subsequently, when validating the model with the Test data set, the ROC curve was calculated and the AUC was measured to assess its performance. Figure 12 shows the ROC curve, which had an AUC of 0.9986. The model also achieved an accuracy of 0.9985, a true negative rate of 99.85%, and a true positive rate of 99.93%.

**Figure 13:**
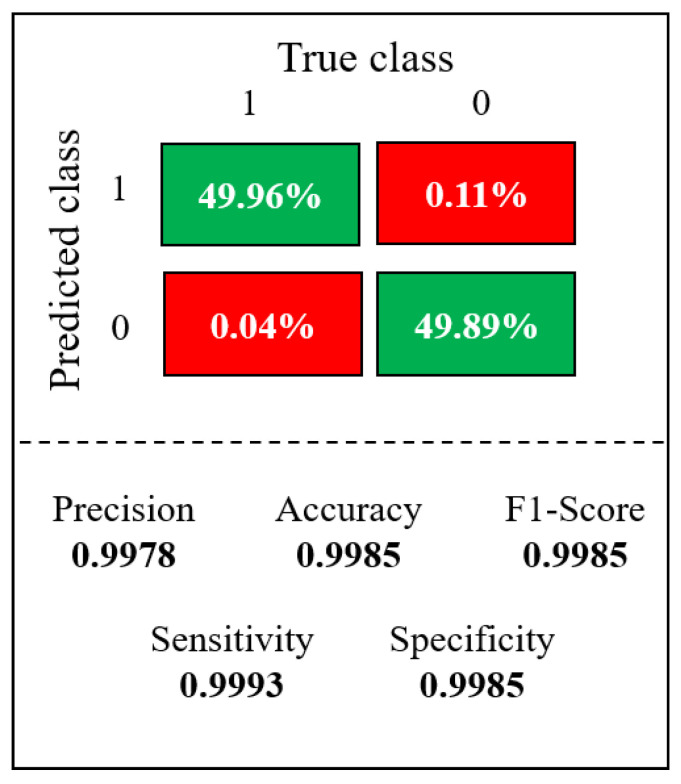
Confusion matrix performance in the test dataset, top zone: green = true negative, red = false negative, and bottom zone: green = true positive, red = false positive.

The confusion matrix shown in Figure 13, from the final model, tested on the blind data set, the model achieved a precision of 0.9978.

**Figure 14:**
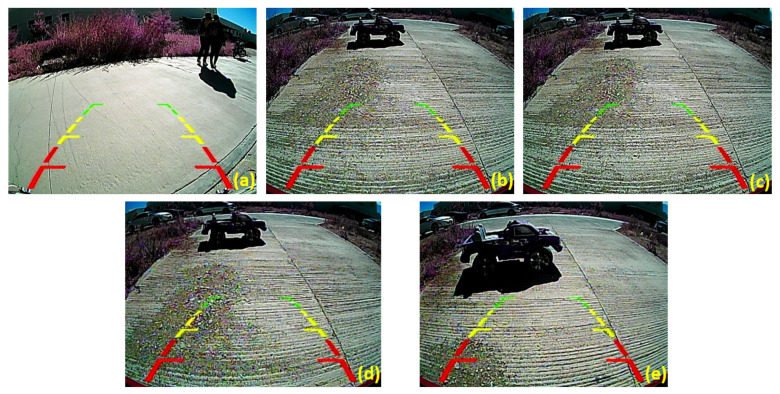
Images from the test data set, the model misclassified: (**a**) Images with pedestrian presence misclassified and (**b**–**e**) images with object presence at different distances in which the model classified as a person.

Based on the results obtained in the confusion matrix (Figure 13), it could be identified that the images shown in Figure 14 corresponded to 0.04% and 0.11% wrong predictions. These values reflect the proportion of incorrect classifications the model made about the total predictions.

Table 3 provides a detailed overview of the data where the model made incorrect predictions. In this Table 3, the actual labels of the images presented in Figure 14, as well as the probabilities assigned by the model to the pictures, are presented, along with their corresponding outputs. This arrangement allows us to conduct a comparative analysis between the actual values and the values predicted by the model, understanding the results of the confusion matrix to identify where the model was wrong.

The results of the prediction of the four images with their respective distances are shown to follow up.

### 3.3. Prediction Test with New Data

**Figure 15:**
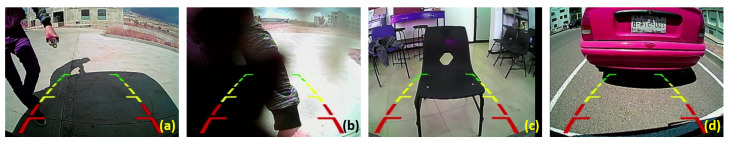
Input images: (**a**) Image with partial presence of a pedestrian, (**b**) presence of a pedestrian very close to the camera, (**c**) presence of a chair with its respective distance, and (**d**) presence of a vehicle.

Additional data were acquired and examined to evaluate the model’s performance and accuracy further. Figure 15 shows a selection of the images subjected to analysis. At the same time, Table 4 presents additional information, where the distances captured by each sensor about the corresponding images and the results obtained from analyzing the pictures with their respective distances are found.

Following the evaluation of the data collected, Table 4 also shows the predictions generated by the model, accompanied by the probabilities derived from the analysis of the content of the input images.

## 4. Discussion

The present study performed raw data acquisition, consisting of distance measurements in centimeters using ultrasonic sensors and reverse camera image captures. The acquired image dataset comprised a wide range of scenarios, including fully visible pedestrians, pedestrians subject to partial occlusion, and pedestrians with no pedestrians. This variety of settings generates a dataset, effectively capturing a broad spectrum of real-world situations. Compared to publicly available datasets such as INRIA and Citypersons [56], the dataset obtained in this research only includes reverse camera images in real driving situations. The research results show the effectiveness of the proposed model for pedestrian detection in vehicle reversing, even in cases with occlusion in the images. It is important to note that, in some previous studies, the authors have used datasets with scenarios dissimilar to everyday driving.

Furthermore, an essential observation in some related works is that pedestrian detection had been performed using only cameras. However, it has been shown that the fusion of sensor information with cameras improves accuracy and decreases detection time. An example is Melotti’s research [57], where LiDAR and camera information were fused using 2D- and 3D-CNN for pedestrian detection. Some recent studies have implemented pedestrian detection using CNN. Table 5 shows the results obtained by other researchers who have used CNN proposals, information fusion, and datasets created or modified by them for pedestrian detection. Also included in Table 5 are the results of real-time detection and implementation of pedestrian detection systems.

Although all current techniques effectively detect pedestrians in a frontal position, only some address the challenge of pedestrian detection in reversing situations using a rearview camera. Among the few investigations found in the literature, as seen in Table 5, the work by Tadjine et al. in 2012 stood out. This study proposed the implementation of a fisheye camera in the rear of a vehicle to detect objects and pedestrians. This task used sliding window techniques, HOG, and SVM as a classifier algorithm. This implementation achieved a sensitivity of 90%, reflecting the system’s ability to detect positive cases, in this case, pedestrians. On the other hand, Matsui et al. [58] investigated pedestrians at the rear of the vehicle using only the vehicle’s ultrasonic sensors. Comparing four sensors (two central and one in each corner)—although they did not use artificial intelligence algorithms—they reported distances and detection percentages. They suggested the detection of children, women, and adult males. They concluded that ultrasonic parking systems can effectively prevent backup accidents involving pedestrians and that combining them with other technologies, such as cameras, could improve safety. The research proposal by Wang et al. [31] did not provide information on the system’s efficiency; however, they gave a detection time of 2.415 ms.

Two significant observations stand out from the results of these studies regarding backward detection. First, the system proposed in this research combines cameras and ultrasonic sensors, unlike previous research that individually addresses camera-based or sensor-based solutions. Second, this study combines information to develop a more robust system, taking advantage of the potential of both one- and two-dimensional CNNs and obtaining an AUC of 99.86% in pedestrian detection. The results obtained in this research also provide detailed information on the performance of the proposed model. The results presented in related research on pedestrian detection in reverse are inferior and need more information on the effectiveness of their systems, which further strengthens the contributions of this research work.

**Figure 16:**
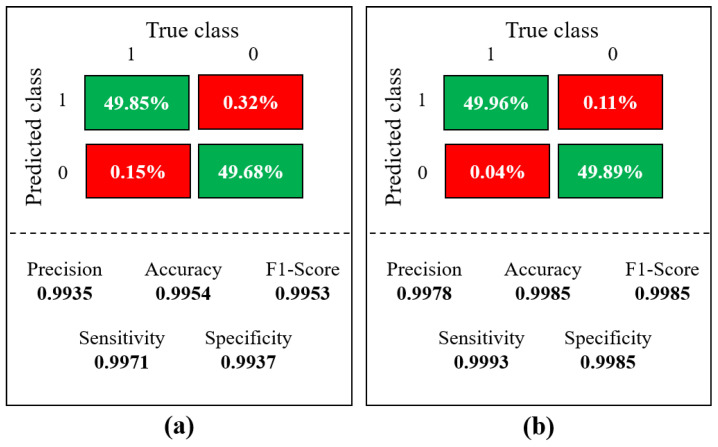
Comparative confusion matrices: (**a**) Images-only vs. (**b**) Images and backward ultrasonic sensor fusion.

Furthermore, a comparative analysis of the model when evaluating images independently, as opposed to the integration of pictures and information from sensors, was conducted. We calculated the confusion matrix for this analysis and used its results to determine evaluation metrics. The performance outcomes of the model are presented in Figure 16. In Figure 16a, the performance when analyzing only the images is showcased. In contrast, in comparison with Figure 16b, a lower commission is observed, accompanied by an increase in the error percentage. Specifically, a false positive rate of 0.32% and a false negative rate of 0.11% are recorded in this scenario.

The results from the fusion of information from the camera and sensors (Figure 16b) display a reduction in the error rate percentages. Therefore, it can be said that using sensors in conjunction with the reverse camera enhances the precision of pedestrian detection.

**Figure 17:**
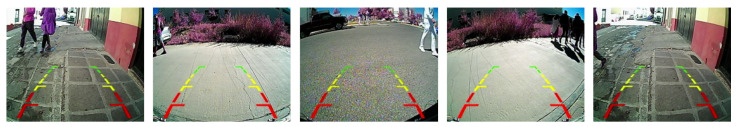
Images that were misclassified.

Figure 17 shows some of the images misclassified by the model when using the image-based analysis alone. However, when distance data from the sensors were integrated, these images were correctly classified.

On the other hand, considering the prediction results of our model shown in Table 4, the presence of additional objects, such as chairs, introduces difficulties to the model in terms of its predictive capability. These difficulties arise due to the limited availability of images in the database containing such objects. Similarly, the images illustrated in Figure 14 are derived from evaluating the model using the test data set but present inaccurate predictions. By examining these images, standard features are identified; specifically, four of the pictures share the same object. From this, it can be inferred that the model failed to learn the distinguishing features of that particular object correctly.

Nevertheless, it is possible to solve these prediction errors by extending the database and subsequently retraining the proposed model, improving its learning capability. Furthermore, given the one-dimensional nature of the data analyzed, using a 1D-CNN architecture is the ideal choice. This selection is further justified by the tendency of the 1D-CNN architecture to incorporate fewer parameters compared to its 2D-CNN counterpart, simplifying the training process and mitigating the risk of overfitting. In addition, the processing time associated with the data type in question is reduced during training and model evaluation.

## 5. Conclusions

In conclusion, pedestrian detection ensures road safety in urban and rural environments. This research explored reversing cameras and ultrasonic sensors to detect pedestrians by fusing two data types, proposing a methodology for 2D and 1D CNNs. The results obtained with our method show that information fusion can achieve high accuracy in pedestrian detection in everyday driving environments. The proposed model achieved an accuracy of 99.85% and an AUC of 99.86% by using two data types. This strategy significantly improved pedestrian detection at the vehicle’s rear, demonstrating the need for more than just images for this task. Furthermore, this research outperformed methodologies in the literature for detecting pedestrians at the car’s rear.

Existing methods are based on specific scenarios using pre-existing datasets. However, this study aims to contribute to developing a new dataset, which can be used as a reference for creating or applying other techniques to solve the problem of pedestrian detection at the rear of the vehicle, thereby reducing the number of reverse driving accidents that may occur.

It is a fact that technology and security systems are becoming increasingly advanced; hence, there is a need to implement these types of systems, while considering the sensors that the vehicle already has. In future work, we plan to expand our database by adding more scenarios, including extreme weather scenarios, as well as more instances in the “non-pedestrian” images, so that the model does not generate false negatives, to make the proposed model more robust, and to develop a pedestrian detection system that takes into consideration all variables that may affect or limit detection capability. These variables will cover a broad range to ensure the effectiveness and reliability of the system.

## Figures and Tables

**Table 1:** Hyperparameters for training architectures.

Hyperparameter	1D-CNN	Inception V3
Input	1×5	299×299
Activation functions	ReLu	ReLu
Epochs	1000	1000
Optimizer	Adadelta [54]	GradientDescent [55]
Number of convolutional layers	3	48
Batch size	64	32
Kernels	3, 2, 1	5×5, 3×3, 1×1
Filters	128, 32, 16	32, 64, 128
Loss	binary_crossentropy	cross_entropy
Output function	Sigmoid	Softmax
Number of classes	2	2
Dense layers	4 of 128, 64, 32, 16 neurons	1 layer of 1024 neurons

The hyperparameters of 1D-CNN were determined on an experimental basis.

**Table 2:** Training and validation results for each value of *K* in the cross-validation.

*K*	Train Acc	Train Prec	Train Rec	Train Spec	Train F1	Val Acc	Val Prec	Val Rec	Val Spec	Val F1	Val AUC
1	0.9641	0.9601	0.9699	0.9568	0.9642	0.9854	0.9854	0.9717	1.0000	0.9709	0.9857
2	0.9950	0.9949	0.9951	0.9949	0.9949	0.9994	0.9994	0.9993	0.9996	0.9993	0.9994
3	0.9985	0.9988	0.9980	0.9989	0.9984	0.9994	0.9994	0.9993	0.9996	0.9993	0.9994
4	0.9998	0.9995	1.0000	0.9996	0.9998	0.9999	0.9999	0.9998	1.0000	0.9998	0.9999
5	0.9988	0.9985	0.9991	0.9984	0.9988	0.9996	0.9996	0.9991	1.0000	0.9991	0.9996
**Mean**	**0.9912**	**0.9904**	**0.9924**	**0.9897**	**0.9912**	**0.9968**	**0.9968**	**0.9939**	**0.9998**	**0.9937**	**0.9968**

Acc = Accuracy, Prec = Precision, Rec = Recall, Spec = Specificity, and F1 = F1 Score.

**Table 3:** Input data to 1D-CNN and the corresponding model prediction.

ID	Distances	Probability	True Value	Prediction
(a)	[436, 1200, 1193, 1196]	0.99699056	1	0
(b)	[86, 104, 447, 1204]	0.01227760	0	1
(c)	[392, 1206, 1200, 1147]	0.00966980	0	1
(d)	[1201, 1078, 107, 1084]	0.02257097	0	1
(e)	[316, 1002, 112, 675]	0.01505166	0	1

The actual value “1” indicates the presence of pedestrians in the image, and “0” indicates the opposite: the missing pedestrians.

**Table 4:** Table of input distances corresponding to the images (ID) shown previously.

ID	Sensor 1	Sensor 2	Sensor 3	Sensor 4	True Value	Probability	Prediction
(a)	89	103	1200	1200	“1”	0.6581990	1
(b)	23	12	35	1200	“1”	0.9653548	1
**(c)**	**1200**	**120**	**135**	**1200**	**“0”**	**0.6395127**	**1**
(d)	190	200	193	199	“0”	0.0151797	0

**Table 5:** Results obtained from proposed work from past research also using CNN and fusion of sensor and camera information.

Title	Technique	Features	Validation Metrics	Results	Front or Back Detection
Vehicle pedestrian detection method based on spatial pyramid pooling and attention mechanism [15] (2020)	YOLOv3-promote	Images	mean Average Precision (mAP) F1 score	91.4% mAP 83.2% F1 score	Front
Pedestrian detection under partial occlusion by using logic inference, HOG, and SVM [23] (2019)	Histogram of Oriented Gradients Support Vector Machine Logic Inference	Images	AUC Error Rate vs. FPPI	Occlusion AUC 20% 98.39% 30% 97.15% 40% 95.41% Error Rate = 64% in 10${}^{-1}$	Front
A part-aware multi-scale fully convolutional network for pedestrian detection [25] (2021)	Part-Aware Multi-Scale Fully Convolutional Multi-Scale Network (PAMS-FCN)	Images	AP log-average miss rate ($MR^{-2}$)	67.2% AP 47.2% $MR^{-2}$	Front
Deep learning approaches on pedestrian detection in hazy weather [19] (2020)	Simple-YOLO, VggPrioriBoxes-YOLO MNPrioriBoxes-YOLO	Images	AP Precision (P) Recall (R)	AP 62.7%, 80.8% and 86.6% P 76.8%, 85.1% and 84.1% R 70.2%, 84.1% and 89.3%	Front
Multimodal deep-learning for object recognition combining camera and LiDAR data [57] (2020)	3D deep-neural network (PointNet) 2D InceptionV3	Image RGB, Depth maps, and Point clouds	Average F-score	97.22%	Front
Object detection and classification using a rear in-vehicle fisheye camera [30] (2012)	Sliding windows Histogram of Oriented Gradients Support Vector Machine	Images	Sensitivity	90%	Back
Pedestrian detection during vehicle backing maneuvers using ultrasonic parking sensors [58] (2020)	Does not use ML algorithms	Distances	Areas and distances	The maximum detection distance ratio: child (32–84%) the adult woman (78–102%) adult man (97–102%)	Back
Novel obstacle detection and tracking system using fisheye vision [31] (2020)	Consensus-based Matching and Tracking (CMT)	Images	Processing time	2.415 ms	Back
**Our Research**	**InceptionV3** **1D-CNN**	**Images** **Distances**	**Precision** **Accuracy** **F1-score** **Sensitivity** **Specificity** **AUC**	**99.78%** **99.85%** **99.85%** **99.93%** **99.85%** **99.86%**	**Back**

## Data Availability

Not applicable.
